# In Situ High-Pressure Synthesis of New Outstanding Light-Element Materials under Industrial P-T Range

**DOI:** 10.3390/ma14154245

**Published:** 2021-07-29

**Authors:** Yann Le Godec, Alexandre Courac

**Affiliations:** 1 Institut de Minéralogie, de Physique des Matériaux et de Cosmochimie (IMPMC), Sorbonne Université, UMR CNRS 7590, Muséum National d’Histoire Naturelle, IRD UMR 206, 75005 Paris, France; alexandre.courac@sorbonne-universite.fr; 2 Institut Universitaire de France, IUF, 75005 Paris, France

**Keywords:** high pressure, synthesis, in situ, X-ray diffraction, synchrotron

## Abstract

High-pressure synthesis (which refers to pressure synthesis in the range of 1 to several GPa) adds a promising additional dimension for exploration of compounds that are inaccessible to traditional chemical methods and can lead to new industrially outstanding materials. It is nowadays a vast exciting field of industrial and academic research opening up new frontiers. In this context, an emerging and important methodology for the rapid exploration of composition-pressure-temperature-time space is the in situ method by synchrotron X-ray diffraction. This review introduces the latest advances of high-pressure devices that are adapted to X-ray diffraction in synchrotrons. It focuses particularly on the “large volume” presses (able to compress the volume above several mm^3^ to pressure higher than several GPa) designed for in situ exploration and that are suitable for discovering and scaling the stable or metastable compounds under “traditional” industrial pressure range (3–8 GPa). We illustrated the power of such methodology by (i) two classical examples of “reference” superhard high-pressure materials, diamond and cubic boron nitride c-BN; and (ii) recent successful in situ high-pressure syntheses of light-element compounds that allowed expanding the domain of possible application high-pressure materials toward solar optoelectronic and infra-red photonics. Finally, in the last section, we summarize some perspectives regarding the current challenges and future directions in which the field of in situ high-pressure synthesis in industrial pressure scale may have great breakthroughs in the next years.

## 1. Introduction

Achieving sustainable growth on a finite planet is a challenge of this century. The needs of societies are changing rapidly and the role of new smart materials in ensuring such transformations is paramount. This challenge requires new kind of materials and, for this, new unexplored dimensions for materials synthesis. As shown by the pioneering study of the Nobel prize-winning Bridgman [1,2], «pressure» opens up an additional dimension, both for the synthesis of new classes of materials with outstanding properties, and for «pressure tuning» physico-chemical features to understand the relationships between structure and the resulting properties. This new dimension has generated great enthusiasm recently because the changes in chemical bonds and properties induced by pressure surpass those due to temperature or even chemical substitution. Hence, the pressure can completely overpass the expectations of traditional chemistry and the potential of high-pressure synthesis is thus immense.

From a fundamental point of view, as an intensive thermodynamic parameter, pressure has a tremendous effect on materials synthesis. Shear stresses, produced during strongly non-equilibrium (and non-hydrostatic) compression, can also be interesting for materials design, although they do not allow getting reliable well-crystallized solids and have not been studied so far in situ using large-volume apparatuses. Pressure can play a crucial role in: (1) preventing the decomposition of thermally unstable chemical precursors; (2) enhancing chemical reactivity and hence decreasing synthesis temperature or reaction time; (3) strengthening chemical bonds and promoting new dense crystal structures.

Such modifications have proven promising in the formation of a large variety of new inorganic compounds with outstanding properties, which are currently unattainable through any other conventional methods. High pressure as an effective industrial technology emerged in the 1950s of the past century after the synthesis of artificial diamond [3,4]—the hardest known material—and the discovery of superhard cubic boron nitride c-BN [5], synthesized a couple of years later. Since the success of these pioneering studies, high-pressure synthesis promoted a large number of new classes of materials with outstanding properties, recoverable at ambient conditions. The most remarkable are: (1) new superhard phases [6] with electrical conductivity like c-BC_5_ [7] or even with non-carbon composition—like nanostructured phases of c-BN [8,9] and high pressure boron allotrope γ-B [10]; (2) new multiferroic materials (like BiMnO_3_, BiAlO_3_, BiGaO_3_, BiFeO_3_ [11]; (3) new thermoelectric compounds (silicon clathrates [12] or CoSb_2_._75_Te_0_._20_Sn_0_._05_ [13]); (4) original semiconductors (e.g., open-framework silicon allotrope Si_24_ [14]); (5) novel energy storage materials (e.g., magnesium carbides [15,16] or polymerized CO compound [17]); and (6) new superconductors (Ba-based silicon clathrates and clathrate-like carbon-framework analogues [12,18,19] or a series of new iron (nickel)-based compounds [20]). All these materials, which are impossible or very difficult to synthesize by conventional-chemistry methods, have great values for potential technological applications.

In the past century, the design of a new material using high pressure and high temperature (HP-HT)—like artificial diamond—required many years of intensive experimental work of academic or industrial research institutes. Nowadays, a couple years of research can be sufficient to propose and optimize a new high-pressure material with its full physical characterizations. If the characterization methods of materials science are basically the same, what has changed during the last years is the possibility to probe the transformations in the chemical systems by in situ X-ray probes using synchrotron radiation such as X-ray diffraction (XRD). Actually, synchrotron sources are necessary because X-rays generated by these facilities are characterized by very high flux and brilliance (about 10^13^ and 10^15^ times higher in 3rd and 4th generation synchrotrons respectively than in X-ray conventional laboratory set-ups) that allows X-rays passing through the high-pressure assemblies and to perform X-ray diffraction in real time during HP-HT experiments

Such in situ HP-HT probing overcomes the intrinsic limitations of the last century ex situ ‘cook and look’ time-consuming methodology. The real-time observation of the precursor evolution has become essential. It allows to find (and optimize) the (P,T) conditions required for the synthesis by tackling the mechanisms and the kinetics of structural and chemical changes.

Two directions are actually developing for high-pressure design of new crystalline materials. One consists of exploring very high pressures beyond the industrial limits (P > 20 GPa) in order to reach significantly unusual solids (for example room temperature superconductor [21], or polymerization of CaC_2_ [22]) that would allow understanding the links between advanced properties and crystal structure/composition, which we will not consider in this paper. Another methodological approach, more pragmatic but still promising, consists of the in situ exploration of “traditional” industrial pressure scale for the design of new stable or metastable compounds by also playing with kinetics (e.g., for nanostructuring) and nucleation-growth mechanism for phase selection [23]. Fixing this “industrial limit” to 20 GPa, what we are actually doing in this review, is quite challenging, but it remains industrially accessible, for example, in advanced technology of production of sintered nanodiamond with advanced mechanical and optical properties [24]. This “industrial limit” justifies our focus on large volume presses technologies (designed for in situ X-ray diffraction) where sufficient amounts of a high-pressure material can be produced and, hence, commercialized.

Additionally, to meet the challenge of sustainable growth it is obvious that the new materials designed by high-pressure synthesis should be composed by the most immediately available chemical elements in the Earth’s crust. It is interesting to note that more than 99% of the atoms that we can find on the surface of the Earth are necessarily included in a shortlist of 10 light elements which are (approximate % by weight) [25]: oxygen (46.6%), silicon (27.7%), aluminum (8.1%), iron (5.0%), calcium (3.6%), magnesium (2.1%), sodium (2.8%), potassium (2.6%), titanium (0.6%), and carbon (0.2%). That is why in this review, we aim to focus on recent examples of new light compounds (composed mainly with the light elements aforementioned) synthesized recently under high pressure by the assistance of in situ X-ray diffraction with synchrotron radiation.

This paper is organized as follows. In Section 2, we will provide an in-time comprehensive review of the recent advances in the development of high-pressure devices which are adapted to X-ray diffraction in many beamlines of synchrotrons, so that the non-specialist readers can get a clear idea of the methods widely employed at present time. The use of these devices in the last twenty years for exploring new materials has led to new successful in situ high-pressure syntheses of light-element compounds that are presented in Section 3. Finally, some perspectives regarding the current challenges and future directions are given in Section 4. The materials syntheses described in our review principally concern (quasi-) hydrostatic synthesis conditions that allows producing well-crystallized powders and crystals (at high temperatures, melt or supercritical fluid form at each synthesis described).

## 2. Methods

There is a wide range of means to generate static HP-HT conditions for synthesis under extreme conditions (here under “static” we understand those that can be maintained from few minutes to few weeks). Typically, heating does not create significant problems for in situ studies, so we will not discuss it in detail, while creation of high pressure does. The majority of HP devices are based on the same principle: the force needed to generate high pressure is created by the application of a primary pressure, from a few tens to several thousand bars, on the piston’s surface of a hydraulic or pneumatic press. Applied on (at least) 2 anvils, this force is transmitted on the experimental assembly, including a gasket, the chemical precursors, and most often a pressure-transmitting medium. The surface of this assembly is much smaller than the surface of the piston on which the primary pressure is applied, this has the effect of generating a pressure multiplied on the sample according to the “principle of intensification”. If this principle is general, however, this type of device can fall into two main categories, according to the sample volume brought under high pressure: diamond anvil cells (DAC) and large volume devices.

### 2.1. Diamond Anvil Cell

The DAC is a compact opposed-anvils device, used as early as 1959 [26], and typically measuring only ~60 mm in diameter for a weight of a few hundred grams (more details and schematic drawings can be found in ref. [27]). It can generate pressure of around 100 GPa routinely and at most 600 GPa (with the risk of irreversible breakage of diamonds at decompression). The principle of a DAC is simple: it involves compressing the sample, previously placed in a hard metallic gasket (general case), between the truncated ends (culets) of a pair of opposite diamonds. For this, one of the diamonds is placed on the fixed body of the cell, in which a moving piston is adjusted and driven by a primary force, together with second mounted-on diamond. The experimental volume is therefore reduced to the hole in the metallic gasket (typically around a hundred microns in diameter or 1/3 of culet diameter), in which the sample, the pressure gauge, and a pressure transmitting medium are housed. The pressure is typically measured by visible fluorescence of a known standard (e.g., ruby [28]); alternative methods can also be used (e.g., P-V equations of state or piezospectroscopic methods [29]). The heating of the sample inside DAC (unfortunately, together with diamonds) may be provided by resistive furnaces surrounding the diamonds for temperatures below 1200 K. To reach higher temperatures (up to 7000 K), mainly the beam of a powerful infrared laser (YAG or CO_2_) is used. Temperature measurement is usually done by thermocouple (for resistive heating) or optical pyrometer (above 1500 K). The main advantages of a diamond anvil cell are, in addition to the ability of obtaining very high pressures and temperatures, the transparency of the diamonds which, with its small size, allows the device to be adapted to a wide variety of structural and spectroscopic techniques. In particular, DAC is widely used for academic in situ studies by X-ray diffraction of synchrotron radiation on the synthesis of new room-temperature superconducting materials (for example, carbonaceous sulfur hydride above 200 GPa [21]) or novel high-energy-density materials beyond 40 GPa (for example LiN_5_ [30]).

Nevertheless, DAC also presents a major drawback for synthesis experiments because the sample volume studied (and especially recovered) is tiny (of the order of 10^−5^ mm^3^), which in principle prohibits any very advanced physical and chemical characterization. Additionally, no reliable study of interactions in mixtures and construction of binary phase diagram is possible. Obtaining very high temperatures under high pressure is particularly difficult, because of the unavoidable high thermal gradients even in the case of very small sample volumes. In addition, chemical reactions (under such extreme pressures and temperatures) with the metallic gasket or the pressure-transmitting medium, as well as carbon diffusion from diamond, are quite frequent, making the exploitation of the results very complicated. Finally, the use of precursor samples of fine chemistry and/or of overly complex properties (e.g., solvent synthesis) is technically quasi-impossible in DAC. Any industrial perspective of a DAC synthesis is still challenging to consider. Thus far in situ DAC synthesis research, although of great fundamental interest, is limited to “exotic” materials under very high pressures, beyond the “industrial limit” situated at ~20 GPa.

### 2.2. Large Volume Devices

Large volume presses (LVP) are already widely used in the industrial environment, and they are constantly developing in several countries, particularly in China at the present time. LVPs (more details can be found in reviews [31,32]) are able to compress few-millimeter samples (and larger) to pressures beyond 1 GPa. Some of these devices are only suitable for ex situ experiments because their sample/reaction-volume environment is opaque to X-rays (piston-cylinder, Belt, Bridgman or Drickamer apparatus), whereas multi-anvil and Paris–Edinburgh cell (Cf. Section 2.2.3) devices have been mainly (re)designed and used during these last few years in conjunction with in situ X-ray diffraction studies at synchrotron facilities.

#### 2.2.1. Multi-Anvil Devices

Multi-anvil presses (MAP) are generally classified according to the number of anvils they use and therefore depend on the shape of the polyhedral space in which the pressure transmitting medium is inserted. Thus, the tetrahedral, cubic, and octahedral presses have 4, 6, and 8 anvils, respectively. When the anvils are brought together, they apply a more “hydrostatic” pressure (in fact, in contrast to uniaxial DAC compression, in the case of MAP the compression is homogeneous in all three space directions) on the pressure-transmitting medium. This allows generating the highest pressure inside the sample assembly (typically of cylindrical shape), i.e., in the reaction volume where synthesis occurs.

The anvils can be driven either by an individual ram or by using guide blocks and applying uniaxial pressure. MAPs generally use automatic pumps to control primary pressure on one or more hydraulic rams which will effectively compress the anvils. Since the force must be sufficient, given the size of these anvils, large presses weighing several tons must be used. The first multi-anvil devices were developed in the middle of the 20th century with pioneering Hall’s tetrahedral MAP in 1958, with four hydraulic rams [33]. Later, von Platen developed a cubic device with spherical anvils submerged in oil [34], while Kawai was the first to propose an octahedral device [35]. The Kawai-type press was then greatly improved, first by introducing a new two-stage compression [36]: the octahedral pressure medium was inserted into an assembly of eight tungsten carbide (WC) cubes pressed by six primary anvils. In 1990, Walker et al. [37] simplified the first compression stage by introducing the removable “hatbox design”, allowing ordinary presses to be converted into MAPs. Such a type of multi-anvil module is called Walker’s module. The two-stage compression design and Kawai octahedral HP cells can also be used in cubic devices of the DIA type devices, i.e., when the force on the WC-cube assembly is applied by six primary anvils.

**Figure 1:**
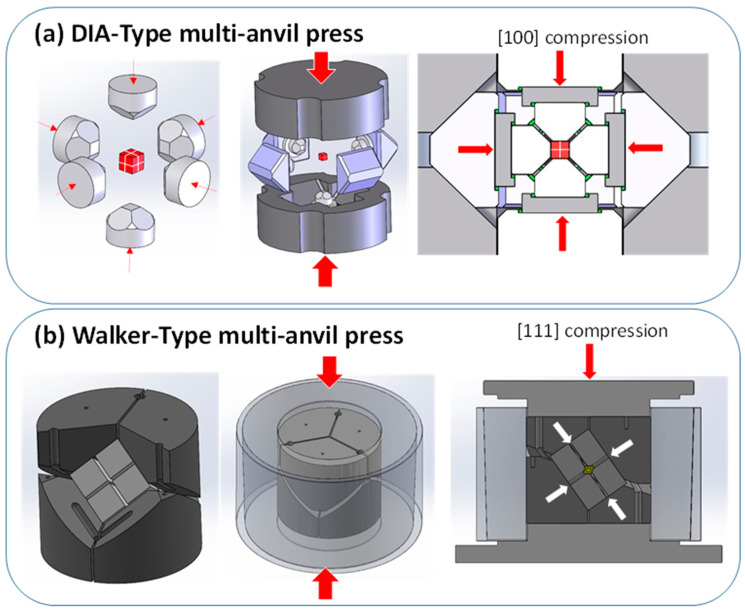
(**a**) Schematic view of the DIA geometry, in which the compression of the primary anvils takes place along the [001] directions of cubic assemblage of secondary WC cubic anvils. (**b**) Schematic view of the Walker geometry, in which the primary anvils are inserted in a cylindrical vessel and the primary compression happens along the [111] direction of the WC-cubes secondary-anvil assembly.

Nowadays, the most commonly used MAPs are DIA and Walker types. Figure 1 shows a schematic view and the differences of both press geometries.

**Figure 2:**
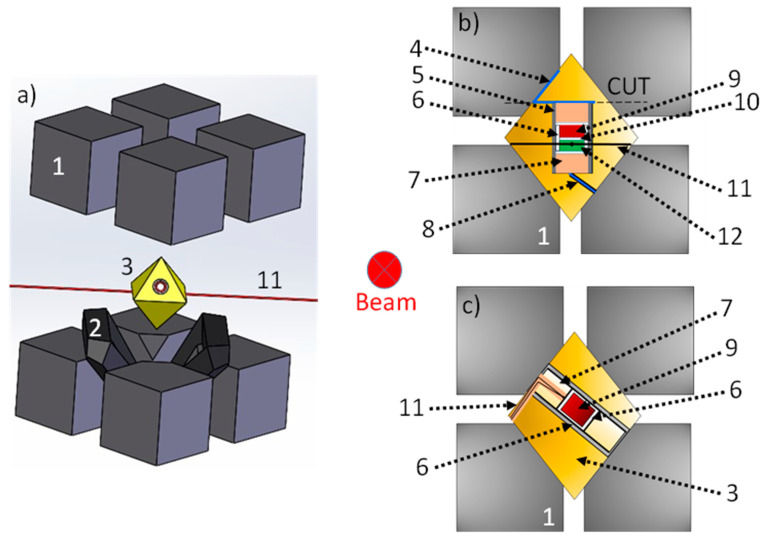
(**a**) Schematic view of the octahedral pressure medium and how it is inserted in the Kawai-type octahedral cell, made of eight truncated WC cubes. (**b**) Schematic view of the typical multi-anvil cell used during EDX and CAESAR experiments on PSICHE beamline at SOLEIL synchrotron. (**c**) Schematic view of the typical multi-anvil cell used during ADX experiments on ID06 beamline at SOLEIL synchrotron. (1) Partial view of WC cube. (2) Gaskets. (3) Octahedral pressure medium. (4) Molybdenum foil. (5) TiB_2_ heater. (6) Hexagonal boron nitride (h-BN) capsule. (7) ZrO_2_ rods. (8) Molybdenum powder. (9) Sample. (10) Hexagonal boron nitride (h-BN) cap. (11) Thermocouple in Al_2_O_3_ tubing. (12) MgO/h-BN powder pressure calibrant.

Both MAP have double anvils stage arrangement: primary and secondary anvils. Convention is that we count the stages from “outside”, which means that the outer six hard-steel anvils are called primary anvils, whereas the eight cubes are secondary anvils. The Walker’s module has a cylindrical cavity filled with the six hard steel primary anvils, three facing up and three facing down. These 6 anvils form a cubic cavity dedicated for the secondary anvils. On the contrary, in the DIA module, there is no cylinder and the first stage anvils consists of four independent moveable equatorial anvils and bottom and top anvils (Cf. Figure 1) that apply forces perpendicular to the cube’s surfaces. Hence, the main difference between the DIA and the Walker MAP module stands in the orientation of the cubic cavity in relation to compression axis, i.e., the [111] direction of the cube in the Walker module, and the [100] in the DIA one. The second stage of compression in both the DIA and Walker MAP is a set of eight cubes with truncated interior corners (Cf. Figure 2).

These truncations form an octahedral assembly cavity. The usual material of this second stage is tungsten carbide (WC), although the use of cubic boron nitride (c-BN) or sintered diamond (SD) cubes is also possible, although more expensive. Only two of these eight cubes have electrical contacts with primary anvils. These cubes are located diagonally opposite to each other and are in contact with furnace ends of the sample assembly. The truncations are directly in contact with the octahedral pressure medium and by varying their size and hence the size of the octahedron, it is possible to attain different pressures. The assemblies are thus defined by the octahedral edge length (OEL) and the truncation edge length (TEL). The standard OEL/TEL assemblies are: 18/11, 14/8, 10/5, 10/4, 8/3 that can provide the synthetic volumes of 12 mm^3^ at 4–10 GPa, 5–8 mm^3^ at 11–16 GPa, 3 mm^3^ at 17–21 GPa and 1 mm^3^ at 22–26 GPa, 0.5 mm^3^ at 30–35 GPa, respectively.

In both DIA and Walker modules, the sample assembly has thus a cylindrical shape inside the octahedral pressure medium. The usual octahedron is made of magnesium oxide MgO with often small additions of Al_2_O_3_, Cr_2_O_3_, etc. Before using the octahedron in high-pressure experiments, it is often fired above 900 °C during a few hours. This procedure results in harder octahedron, permitting to reach higher pressures. The hole inside the octahedron, dedicated for the sample assembly with furnace and thermocouple, usually passes through two opposite faces of the octahedron. These multi-anvil devices use resistive heating with an internal heater (graphite, chromite LaCrO_3_, TiB_2_, TiC, metallic foil, etc.) inside the octahedron to reach simultaneous high temperature and pressure (Cf. Figure 2 where two typical set-ups are shown). In addition, gaskets, prepared usually from materials with low coefficient of internal friction like pyrophyllite (this particular material has important property of increase of plasticity under compression up to maximum and subsequent decrease), fill the space between two adjacent cubes and provide electrical insulation between the secondary anvils. Temperature can be measured using thermocouple or estimated using electrical power-temperature calibration-curve method. Pressure can be also estimated with prior calibration curves (maximal or mean pressure in the reaction volume vs. applied hydraulic-oil pressure). In both cases of pressure and temperature, the larger cell assures higher reproducibility of calibration curves.

#### 2.2.2. Adaptation of Multi-Anvil Devices to In Situ Synchrotron X-ray Diffraction

The compression efficiency in a DIA apparatus is usually less than in a Walker-type MAP, but DIA module is accessible for in situ synchrotron X-ray diffraction (XRD) while the highly X-ray absorbent steel confinement ring around the anvils of a Walker module makes X-ray diffraction impossible in this configuration. In DIA-type MAP, the four equatorial anvils of the first stage can have a horizontal groove dedicated to incoming and outgoing X-ray beams. That is why DIA multi-anvil presses are widely used in all synchrotrons around the world. Two main X-ray diffraction techniques have been developed with MAP: angle-dispersive XRD (ADX) and energy-dispersive XRD (EDX). Whereas EDX technique is used at polychromatic X-ray radiation and is generally associated with a solid-state detector at a fixed angle (2θ), ADX technique is operated only with monochromatic incident X-ray beam and 2D detector.

High quality crystallographic data can be yielded from the ADX measurements (symmetry, structure, internal coordinates of atoms, and microstructure of crystalline samples, etc.). However, the ADX has also some disadvantages, which can severely limit its use, like for example rather long acquisition time needed (compared to EDX) and requirement of a very high spatial accessibility (which is difficult with MAP’s geometry).

**Figure 3:**
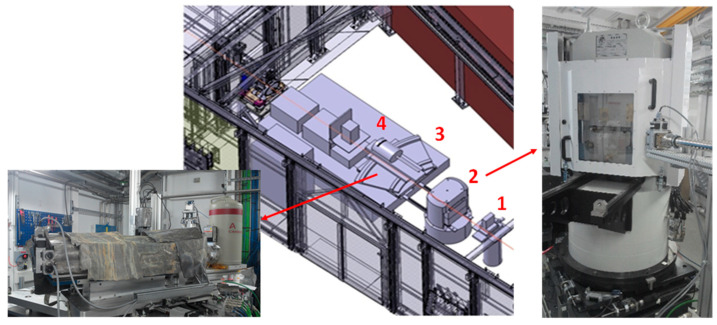
Optics hutch of PSICHE beamline at SOLEIL, a synchrotron X-ray diffraction station (EDX) associated with a multi-anvil press. (1) Input collimating slits. (2) Multi-anvil press. (3) CEASAR system with (4) solid-state germanium detector.

Conversely, EDX does not generally allow a very precise quantitative crystallographic analysis of the sample studied (for example prohibiting any Rietveld refinement of crystal structure) because the relative intensities of the diffraction peaks are convoluted by the emission spectrum of the synchrotron source energy and by the energy-depending absorption (contingent also to gaskets extrusion with (P, T) conditions) along the X-ray beam path. However, EDX has some advantages over the ADX technique. The quality of the new detectors is such that recording an EDX spectrum takes a few μs or ps only. This speed makes EDX a powerful tool for rapid structural analysis (e.g., to detect intermediate phases, which are unstable or exist for a very short period) or to perform kinetics studies on the synthesis of new materials. On the other hand, the decisive advantage of EDX is the ability to obtain diffraction spectra with a point detector without the use of a goniometer. The fixed scattering angle therefore allows efficient use of diffracted X-ray collimators in order to define very precisely the detected area, resulting in the removal of the unwanted diffraction signal from the sample environment. Therefore, high spatial accessibility of the sample is not required and EDX can be applied for in situ experiments with significantly limited access to the sample. This is why this diffraction technique is mainly used for high-pressure experiments in multi-anvils. Figure 3 shows the various elements of a typical synchrotron X-ray diffraction station (EDX) associated with a multi-anvil press.

Figure 3 also allows us to present a third hybrid mode associated with X-ray diffraction: the Combined Angle and Energy-dispersive Structural Analysis and Refinement (CAESAR) method. This technique proposed by Wang et al. [38] is now in operation with MAP at PSICHE beamline of SOLEIL synchrotron in France. It consists of collecting series of one dimensional EDX patterns (intensity versus energy E) as function of diffraction angle 2θ, by varying the position of the detector and the 2 pairs of output slits (Cf. Figure 3) placed behind MAP (i.e., by keeping the same volume of sample investigated with a very small sphere of confusion). Hence, at the end of the acquisition, the entire data set contains all 2θ steps (up to 0.01°) from 0° to 30° and forms a two-dimensional array (2D diagram): Intensity (E,2θ). These intensity data can be then regrouped according to photon energies, providing a large number of angle-dispersive (intensity versus 2θ) patterns, each of which corresponds to a given photon energy. This combination of energy-dispersive and angle-dispersive enables exploring a wider region of the reciprocal space. Thus, the collection and the analysis of CAESAR data (via an algorithm that re-normalizes the intensities) allows finally to perform structural Rietveld refinement, even if performing primarily EDX.

The choice between these three different X-ray diffraction techniques involves significant modifications to the sample environment in an in situ high-pressure multi-anvil press experiment.

For ADX, like at ID06 beamline of ESRF, it is impossible to collimate the diffraction signal of each element on the beam path (Sollers slits are impossible to implement in MAP, due to the limited aperture). Hence, along the beam path, cylindrical amorphous SiBCN X-ray windows and wide amorphous boron epoxy rectangles are inserted into the octahedron and gaskets, respectively. Indeed, when using a monochromatic beam, the flux is considerably reduced. It is thus important to insert non-crystalline and low-absorbing elements along the beam path as to preserve the high quality of the diffraction data. The cross-section of the typical octahedron used in ADX at ESRF is shown in Figure 2c. It indicates the position of the various components of the sample assembly.

For EDX, as compared to ADX, the use of the polychromatic beam ensures higher flux. Additionally, the efficient use of collimators (resulting in the elimination of the unwanted diffraction signal from the sample environment) makes the use of non-crystalline and low-absorption insertions not necessary once the beam is focused on the central part of the assembly.

Additionally, to perform CAESAR acquisition, like at PSICHE beamline of SOLEIL synchrotron, the sample volume probed during the experiment should be identical at every diffraction angle. To ensure this, the cylindrical sample assembly should be placed perpendicularly to the plan of the beam. In this way, the depth of the measured sample is invariant with respect to the azimuthal angle. We typically cut the octahedron into two parts; and the hole hosting the high-pressure assembly is drilled in the cut surface, as shown in the cross-section of the octahedron in Figure 2b.

#### 2.2.3. Paris–Edinburgh Press

The Paris–Edinburgh press is a “two-opposed-anvil-type” large volume device. Developed 30 years ago, this press was designed preliminarily for high-pressure experiments in neutron sources such as ISIS in the United Kingdom [39]. The design idea was to generate maximum force in a minimum of space so that the press could be easily installed on any beamline without performing major work to accommodate a huge device (like multi-anvil presses). The originality of the Paris–Edinburgh press comes from its hydraulic ram, which is a key breakthrough. It has been optimized (by finite element calculations) to reduce both size and weight. The outcome is a ram with a high capacity (from 500 kN to 4500 kN) which weighs only a few kilograms (from 10 to 90 kg depending on the version) and fits into a 40 cm cube. The press is also easily removable, with parts not exceeding 20 kg, which facilitates its handling and transport. This compactness made it possible to adapt the Paris–Edinburgh press in various synchrotrons or neutron sources as well as in many academic laboratories all over the World (Cf. below).

**Figure 4:**
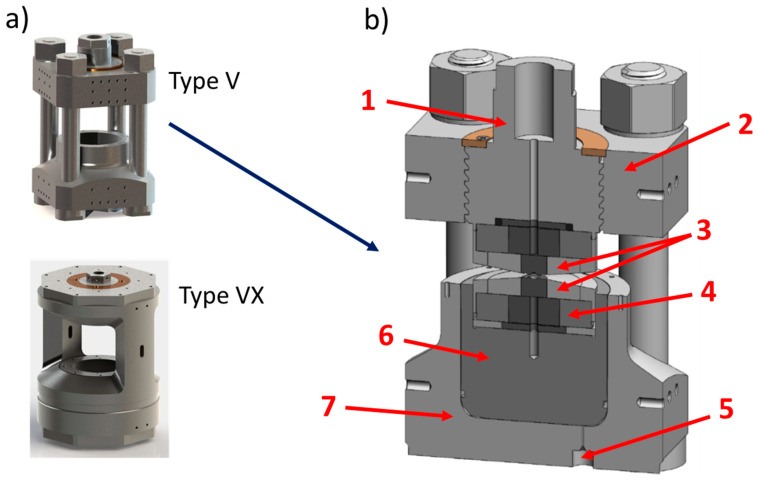
(**a**) The two types of Paris–Edinburgh (PE) press with 4 (type V) and 2 columns (type VX). (**b**) Cross-section of the V3 PE press: (1) breech, (2) top platen, (3) anvils, (4) seat, (5) hydraulic fluid inlet, (6) hydraulic piston, (7) ram.

As shown in Figure 4, depending on the version, the Paris–Edinburgh press can have 2 or 4 columns: generally, notation VX is used to designate a Paris–Edinburgh press with 2 columns (thus allowing a wider angular opening) [40] and V—a Paris–Edinburgh press with 4 columns.

This Figure 4 also shows a sectional view of a Paris–Edinburgh press V3 and allows to simply illustrate its principle of operation: a hydraulic fluid or gas pressure is applied to the base of the piston via a high-pressure generator (automatic compressor, hand pump, etc.) connected to the press ram by a high-pressure flexible tube. The top part of the press is fixed. The piston then pushes a tungsten carbide seat, fretted in a steel part (with a radial stress of 1 GPa), which transmits the force to 2 opposed anvils: the high-pressure part is located between the anvils. These anvils can have several shapes, permitting to provide HP-HT experiments in various pressure ranges. In their standard design, these tungsten carbide (WC) anvils (but for reaching higher pressures, sintered diamond (SD) anvils could be used), also fretted with hard steel, have a circular high-pressure cavity with a quasi-conical profile, dedicated for gasket and sample assembly. The gasket is characterized by the value of its external diameter (ED) and of its central hole (where the sample assembly is housed), corresponding to the flat surface of this high-pressure cavity (FS). Various geometries ED/FS exist. For instance, 12/5, 10/3.5, 7/2.4, and 5/1.5 geometries correspond (with SD anvils) to the maximum reachable pressures of 6, 8, 10, and 17 GPa, respectively [41]. For ex situ or in situ neutrons diffraction studies, gaskets (or pressure medium) are made of baked pyrophyllite. Nevertheless, pyrophyllite absorbs too much X-rays and cannot be used for synchrotron techniques. That is why for in situ X-ray diffraction studies, amorphous boron-epoxy gasket, which is almost transparent to X-rays, is used. Occasionally, an additional Teflon (or PolyEtherEtherKetone PEEK) ring can be positioned around the gasket in order to moderate the gasket flow under compression.

**Figure 5:**
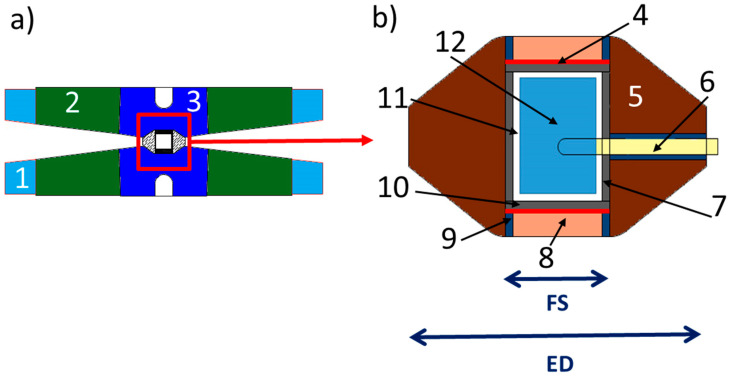
Schematic diagram of two-opposed anvils (**a**) and sample assembly (**b**) of the large volume Paris–Edinburgh press for in situ X-ray diffraction studies (here for ED = 10 mm and FS = 3.5 mm). (1) Cooling device. (2) Steel binding ring. (3) Tungsten carbide WC anvils. (4) Molybdenum disk. (5) Amorphous boron epoxy gasket (pressure medium). (6) Coaxial thermocouple. (7) Graphite heater. (8) Insulating ceramic. (9) Electrical contacts. (10) Graphite disk. (11) h-BN or MgO capsule. (12) Sample.

Inside the gasket, the sample assembly consists of the usual various parts of traditional high-pressure components: sample capsule, cylindrical heater, insulating ceramics, thermocouple, etc. Depending on the experiment’s requirements, these parts can be prepared from various materials. Furthermore, some parts can be either removed or added. Figure 5 shows a basic sample assembly associated with the 10/3.5 geometry.

As in multi-anvil press, temperature is generated by Joule effect (resistive heating). Sample temperature is measured with a coaxial thermocouple, with no correction for the pressure effect on the thermocouple electromotive force. In case of thermocouple failure, the sample temperature can be also well estimated by an established power-temperature relation, which depends mainly on the gasket’s geometry. In off-line experiments, pressure is also estimated with prior calibration curves where the sample pressure is plotted as a function of the controlled applied primary (oil) pressure on the piston.

#### 2.2.4. Adaptation of Paris–Edinburgh Press to In Situ Synchrotron X-ray Diffraction

**Figure 6:**
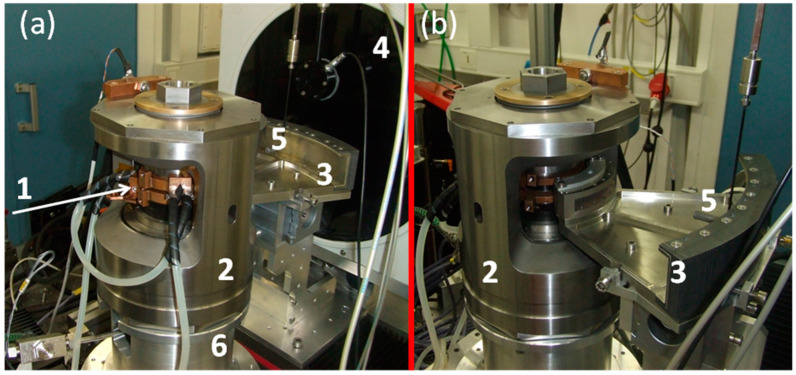
Front (**a**) and rear (**b**) views of the Paris–Edinburgh press installed on the ID27 beamline of ESRF. (1) Incoming beam. (2) Paris–Edinburgh press. (3) Sollers slits system. (4) Image-plate detector. (5) Motorized beamstop. (6) Press-positioning x-y-z motors.

Thanks to its compactness, the Paris–Edinburgh press has been adapted during the last years for a wide range of in situ high (P,T) measurements such as neutron and X-ray diffraction [42,43], extended X-ray absorption fine structure (EXAFS) [44], Compton scattering [45], inelastic neutron and X-ray scattering [46], ultrasonic [47], or tomographic studies [48]. For X-ray diffraction, this press is widely used in almost all synchrotrons in the world and can be adapted to all the diffraction geometries mentioned above in Section 2.2.2: ADX, EDX or CAESAR measurements. In particular, the ADX mode has been largely developed thanks to the exceptional angular opening of the press (140° for a VX3) and the development of an original Sollers slits system [49]. Actually, as shown in Figure 6, in a X-ray diffraction beamline like ID27 of ESRF, the diffracted beam can be easily collimated by a Sollers slit system, installed on translations and allowing alignment of the central slit on the incident beam.

This multichannel collimator consists of two concentric sets of 75 fine slits (WC blades) at 0.8° angle from each other to cover a total angle of 60°. Both arrays are located 50 and 200 mm, respectively, from the sample (centered on the rotation axis of this multichannel collimator). Slit widths of the inner and outer arrays (0.05 and 0.20 mm, respectively) are designed in order to minimize the volume seen by the two-dimensional detector and maximize the signal detected. These slits are mechanically aligned with high precision on a base-plate made of Invar to prevent from any variation of the system dimensions in case of temperature fluctuation in the experimental hutch. By oscillating the slits at a constant speed during data collection, background scattering coming from the material surrounding the sample can be effectively removed and homogeneous angle dispersive data can be collected using a 2D detector (like MAR CCD detectors or hybrid pixel detectors) with large input surface of more than 150 mm diameter. Exposure times are typically between 20 and 100 s (depending on the sample) for a single ADX pattern.

Hence, the Paris–Edinburgh press combined with a multi-channel collimator permits in situ ADX and allows collecting rich crystallographic data sets for samples at extreme pressures and temperatures.

## 3. In Situ Large-Volume High-Pressure Syntheses

As already explained in the previous section, in contrast to DAC experiments, in situ large-volume-press experiments reproduce real industrial conditions for high (P,T) synthesis of new materials. In the following, we will present recent successful in situ high-pressure syntheses of light-element compounds in various systems, performed at synchrotron facilities using multi-anvil or Paris–Edinburgh presses.

### 3.1. Diamond and c-BN

Graphite-to-diamond transformation in graphitic phases of various light-elements (e.g., B-C-N-O) compounds inspired high-pressure synthesis experiments for decades [50,51,52], and for most diamond-like phases, the main hot topic concerned their existence and purity. Graphite, h-BN, and graphitic BC_x_ and BC_x_N phases have been studied in situ in DAC at HP, both at room temperature and HT [53]. Numerous direct phase transformations were observed [54,55], a number of advanced superhard materials were revealed and patented [7,56], and new mechanisms of HP-HT graphite-to diamond transformation developed [57,58]. However, most of the results remain in the field of explorative materials science. They allow understanding deep links between structure, composition, and functional properties at an extended range (e.g., hardness [59,60], superconductivity [61,62], etc.), rather than applying them immediately in industry.

**Figure 7:**
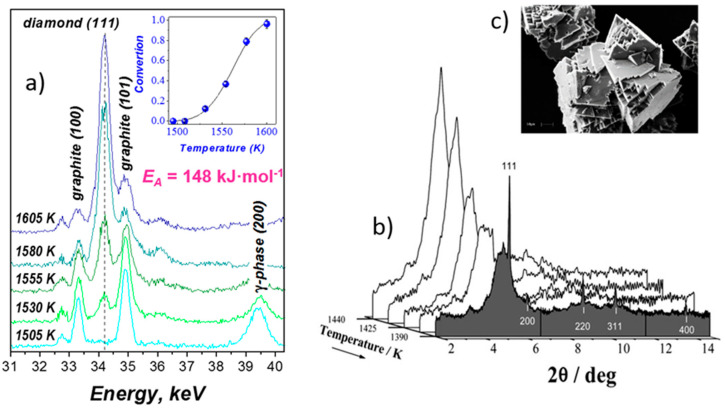
In situ observation of synthesis of diamond and c-BN from their graphitic precursors using a Paris–Edinburgh press at ESRF. (**a**) Graphite-to-diamond transformation in the presence of Fe-Ni-C liquid: energy dispersive X-ray diffraction patterns taken at 5.2 GPa with heating rate 25 K/min [65]. Insert: Kinetic curve of graphite-to-diamond transformation. Temperature changes linear with time, the full curve correspond to ~4 min. (**b**) Crystallization of c-BN from B-N-H supercritical fluid: sequence of angle-dispersive X-ray diffraction patterns taken at 2.1 GPa in the course of cooling the supercritical solution (57 mol.% BN) from 1460 to 1390 K [64]. (**c**) Scanning electron micrograph of as-grown c-BN crystals (2500×) (reproduced from [64] with permission from the PCCP Owner Societies).

At the same time two of them, diamond and cubic BN, are well-known industrial materials whose formation has been thoroughly studied at HP-HT conditions in large volume apparatuses, primary by ex situ since the 1950s, and in situ in the late 1990s methods [63,64,65]. Both materials have a simple diamond-like structure, and, subsequently, a simple XRD pattern that can be easily identified (Figure 7). This fact makes it easy to observe the onset temperature of diamond or c-BN formation in a typical experiment of heating at various chemical systems at stable pressure (in fact, only external oil pressure applied on anvils can be maintained, the sample pressure can—and often does—change with temperature and transformation degree). In situ probing of these materials allowed getting the methodology for constructing the reliable phase diagrams under extreme conditions, as well as understanding the kinetics, mechanism and dynamics of industrial crystallization processes. Some pioneering results of in situ observations of crystallization mechanisms for diamond (in typical ferrous-metal environment) and c-BN (in supercritical B-N-H fluid) under conditions close to industrial are presented in Figure 7.

The data presented in Figure 7a allowed us to study the kinetics of fast crystallization of diamond for the first time; activation energy E_A_ = 148 (47) kJ·mol^−1^, with mechanism implying the 3-D diffusion growth and continuous nucleation [65]. The time scale of full diamond crystallization at ~5 GPa, in the 1500–1600 K range in the presence of Fe-Ni-C solvent was estimated as a few minutes (4 min. correspond to the temperature range of Figure 7a).

Based on the data of Figure 7b, the mechanism of c-BN crystallization in the presence of supercritical B-N-H fluid was established that include equilibria between NH_3_ -intercalated BN disordered layered compound (X), whose role could not be established without in situ observations [64]. This intermediate phase X cannot be recovered at ambient conditions, and this study revealed the impossibility to suggest the correct mechanism of HP-HT crystallization of c-BN without in situ methods. Therefore, studied supercritical synthesis is very efficient for high doping degree of c-BN, e.g., by Si or Be, and recently high-temperature semiconductors (operating up to 875 K) were reported [66].

### 3.2. Boron-Rich Compounds

Boron-rich compounds attracted much attention in the past years for the design of new useful materials combining high hardness, toughness, and resistance to extreme mechanical, thermal and radiation conditions [6,67]. In particular, numerous attempts have been made to fulfill the hardness gap between two reference superhard materials, i.e., polycrystalline diamond (H_V_~80–120 GPa, which is a typical value range of good-quality sintered samples) and two times less harder c-BN (H_V_~40–60 GPa for polycrystalline ingots, also these hardness values for diamond and c-BN can be higher for single crystal faces and nanostructured ingots) [67]. Among icosahedral boron-rich solids, the record hardness has been achieved in the case of high-pressure boron allotrope, γ-B [68,69,70]; while most of the icosahedral boron rich compounds remain at the lower boundary of superhardness, i.e., at ~H_V_ of c-BN [67].

At the same time, such complicated crystal structures with large unit cells have more numerous reflections in XRD patterns [53] as compared to simple diamond and graphite structures, which render them difficult to observe and identify among the diffraction of the HP-cell environment. Numerous explorative in situ studies of the boron-rich part of the B-C-N-O-X system at HP-HT conditions were performed and led (i) to the discovery of new compounds in B-N [71], B-Si, and B-Se systems [72], (ii) to construction of B-O [73], B-N [74], and B-N-O [75] phase diagrams, as well as (iii) to understanding the difficulties of HP synthesis of stoichiometric compounds in corresponding systems [76].

Rhombohedral boron subnitride B_13_N_2_ is a boron rich solid with relatively simple XRD pattern known for icosahedral borides (similar to B_4_C and B_6_O of α-Boron type). It can be synthesized by crystallization from the B–BN melt at 5 GPa [71,76] and is the only stable compound in the system at this pressure [74]. The phase forms reddish crystals, similar to boron suboxide B_6_O, and is predicted to be superhard with H_V_~40 GPa and has the highest incompressibility (or bulk modulus) in “α-Boron family”. At the same time, in contrast to B_6_O, B_13_N_2_ never forms as a single phase in the B-BN system. Only in situ studies and construction of phase diagrams explained this fact.

**Figure 8:**
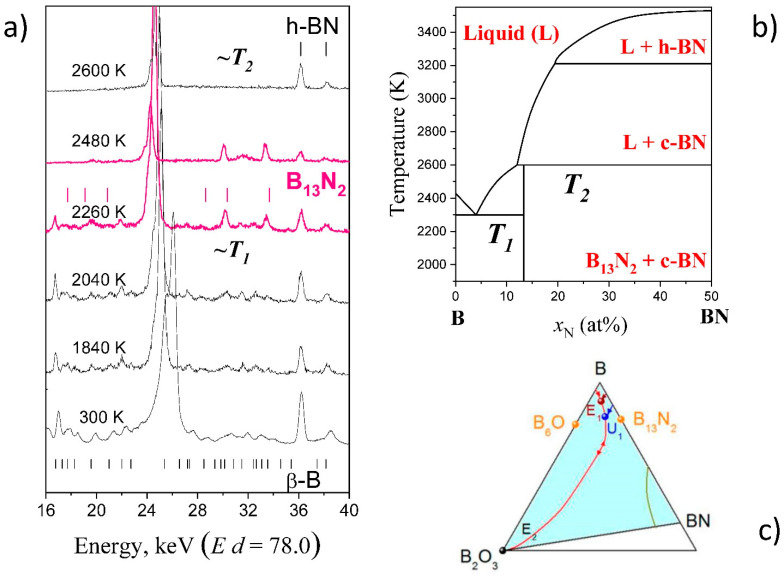
(**a**) In situ observation of synthesis of icosahedral boron-rich compound B_13_N_2_ by chemical reaction of boron (β-B allotrope) with h-BN using a multi-anvil system MAX80 at HASYLAB-DESY [77]. Typical sequence of the energy-dispersive X-ray diffraction patterns taken at 5 GPa in the course of heating of the B-BN mixture to 2600 K [74]. The stars represent the h-BN escape line, while vertical bars show the positions of the phase reflections. The β-B lines disappear between 2040 and 2260 K (T_1_ ~2300 K). The reflections of B_13_N_2_ appear at 2260 K and are observed at temperatures below 2600 K (T_2_ ~2600 K). (**b**) Isobar section (P = 5 GPa) of calculated B-BN phase diagram with temperatures T_1_ and T_2_ as the elements of the calculated phase diagrams [74]. Insert: Large (~200 µm) reddish crystal of B_13_N_2_ in boron matrix [58]. (**c**) Projection of melting phase diagram on the concentration triangle of the B-N-O system [75]. The ternary phase diagram has been established by combining in situ and ex situ data.

Figure 8a shows the in situ synthesis of B_13_N_2_. Initial boron, β-B allotrope, is also observed, but cannot be a priori recognized. The temperatures of transformations at given pressure, T_1_ and T_2_, can be estimated from the pattern sequence (linear heating controlled by thermocouple). In the hypothesis that transformation occurs immediately after the thermodynamic conditions have been achieved (this is often valid at high temperatures and is often used, at least for construction of the first in situ approximation), these values can be used for refinement of the parameters describing the thermodynamic model of liquid and/or solid phase(s). The calculated phase diagram explained the difficulties in synthesis of single-phase samples of B_13_N_2_ (peritectic-type of phase diagram), as compared to B_6_O (simple eutectic type).

### 3.3. Magnesium Carbides

Magnesium carbides have been attempted many times to be placed on the Mg-C phase diagram, which is one of the systems that is interesting for diamond synthesis, both single crystals and B-doped semiconductors. However, the experimental data were controversy [78,79] and inconsistent with thermodynamic consideration [80,81].

In situ studies allowed to shed the light on the crystallographic diversity of magnesium carbides that form at high pressure, i.e., β-Mg_2_C_3_ [16] and Mg_2_C [15], both having p-T-x domains of thermodynamic stability (above 5 GPa) [82]. These high-pressure phases can be recovered at ambient conditions and remain metastable for long time. β-Mg_2_C_3_ is a yellow powder, while Mg_2_C has a dark grey color.

**Figure 9:**
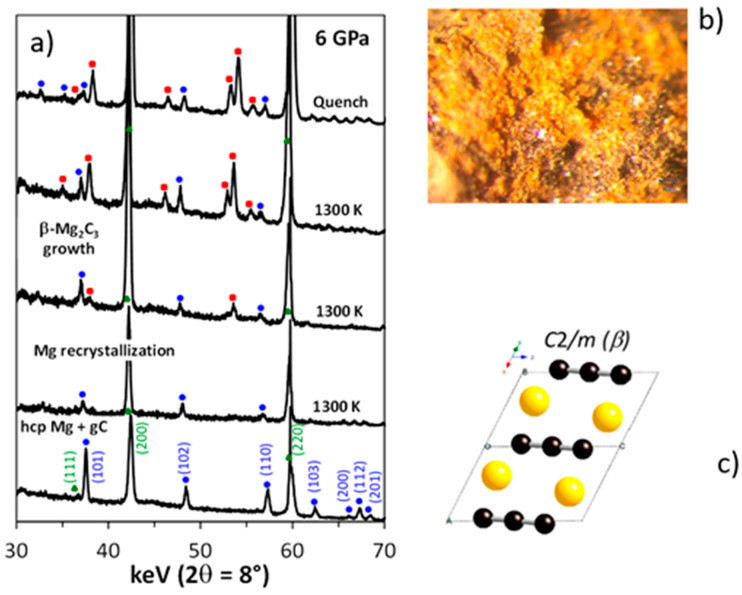
(**a**) In situ observation of synthesis of Mg_2_C_3_ (red circles) by chemical reaction of Mg (blue circles) with powder of glassy carbon using energy-dispersive XRD with white beam of synchrotron radiation in a Paris–Edinburgh-type press at SOLEIL [16]. Green circles show the MgO capsule material. (**b**) Optical microscope picture of as synthesized β-Mg_2_C_3_ powder. (**c**) Monoclinic crystal structure of β-Mg_2_C_3_ resolved by combination of in situ powder XRD and evolutionary structural algorithm USPEX [16]. Yellow balls indicate the position of Mg atoms, while black balls—of C atoms. (Figure adapted with permission from [16]. Copyright 2014 American Chemical Society)

Figure 9a shows the sequence of powder XRD patterns collected at 6 GPa during the heating of a mixture of Mg and C. In contrast to ex situ XRD experiments (powder patterns of multiphase samples), in situ data allowed estimating unambiguously the individual reflections of unknown magnesium carbide phase. Chemical analysis indicated the Mg_2_C_3_ composition with C_3_^4−^ anions, while ab initio evolutionary simulations [83] predicted a number of unit cells for such composition, and one of them was successful, as the monoclinic unit cell was refined using Rietveld analysis of in situ XRD data [16]. Conventional ex situ techniques were very time-consuming to accomplish the characterization of Mg-C system (competing crystallization of low-symmetry phases), even simple cubic antifluorite carbide Mg_2_C was recognized and characterized after in situ HP-HT studies using DAC at HPCAT beamline of synchrotron APS (Chicago, IL, USA) [15]. For monoclinic β-Mg_2_C_3_ phase, the in situ synthesis of large volume samples was crucial [16].

### 3.4. Silicon

Si-I with diamond structure is a low-pressure semiconductive allotrope that passes into metallic Si-II (β-Sn structure), stable above ~10 GPa in all temperature ranges up to melting. A large variety of phases were observed at higher pressures [84,85] and on decompression (Si-III with BC8 structure [86] and Si-XII with R8 structure [87] considered as rhombohedrally distorted BC8), but again, it is not clear whether these phases have a domain of thermodynamic stability. Heating of Si-III gives Si-IV with hexagonal diamond structure [88], which serves an excellent example of synthesis of an advanced material by using high pressure to produce a precursor, not a final product. The diversity of silicon crystalline forms is very promising for both theory and advanced applications in solar cells and photonics [89,90].

Crystal structures of silicon are much simpler as compared to boron, but more complicated than those of carbon. Their observation is easier because Si scattering factor for the X-rays is much higher.

**Figure 10:**
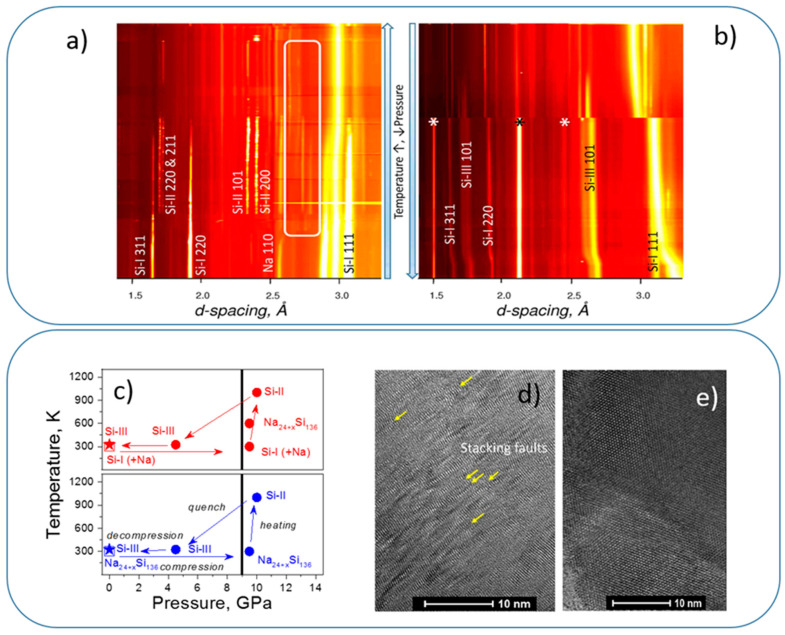
Sequences of XRD diffraction patterns of the Na + Si system at 9.5 GPa during (**a**) heating, and (**b**) quench and decompression using multi-anvil press (ESRF, ID06 beamline) [91]. White contour delimits the reflections that appear during interactions (Na_4_Si_4_ and clathrates), asterisks show the “noise” reflections from the environment that do not move with pressure change (gasket material, etc.). (**c**) Pressure-temperature evolution during Si-III synthesis from the Na/Si mixture (red) in comparison with decomposition of Na_32_Si_136_ clathrate (sII-HP) at high temperature (blue) [91]. Vertical lines indicate the low-limit pressures where Si-III has been observed and can be easily extracted in pure form. (**d**,**e**) TEM images of Si-III sample obtained by direct transformation at HP-HT sample (**d**) and in Na-Si system (**e**). The latter does not show stacking faults, typical for direct-transformation sample [91]. (Figure adapted with permission from [91]. Copyright 2016 American Chemical Society).

In situ HP-HT chemical route to Si-III has been discovered for Na-Si system (initial mixtures: Na + Si; Na_4_Si_4_ + Si or simply Na_30_Si_136_) using both multi-anvil and Paris–Edinburgh techniques [91] (Figure 10a–c). The phase-pure samples have been synthesized at significantly reduced pressure in the Na-Si system at 9.5 GPa by quenching from high temperatures ~1000 K. Pure sintered polycrystalline ingots with dimensions ranging 0.05 to 0.5 mm can be easily recovered at ambient conditions. In situ control of the synthetic protocol, using synchrotron radiation, has allowed observing the underlying mechanism of chemical interactions and phasing transformations in the Na-Si system, as well as pressure-temperature (-time) profiles of synthetic protocols (Figure 10c). The “chemical” samples show better crystal perfection as compared to the samples obtained by direct phase transformation (Figure 10d,e).

### 3.5. Sodium Silicide Clathrates and Open-Framework Allotropes

Sodium silicide clathrates are promising compounds for the design of open-framework silicon allotropes that are predicted to be ideal materials for solar cell applications [92]. Compounds like Na_4_Si_24_ [93] or Na_30_._5_Si_136_ [94] were discovered by exploring high-pressure chemistry of Na-Si system and are the promising precursors for synthesis of open framework Si_24_ and Si_136_ allotropes with quasi-direct bandgap. However, contrary to many other high-pressure syntheses, the Na_4_Si_24_ compound cannot be easily obtained; the in situ studies indicated that it is due to complicated nucleation mechanism [23].

**Figure 11:**
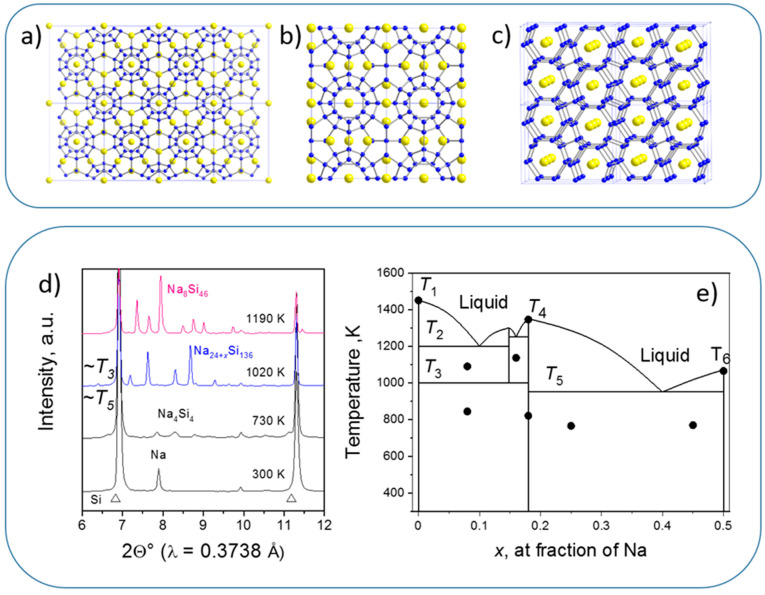
(**a**–**c**) Crystal structures of Na-Si stoichiometric clathrates: (**a**) Na_24_Si_136_—structure II or sII, (**b**) Na_8_Si_48_—structure I or sI, (**c**) Na_4_Si_24_ or NaSi_6_. Blue balls indicate the atomic positions of Si atoms, while yellow balls—of Na. (**d**) In situ XRD data on phase/chemical transformations in the Na + Si mixture (~15 at % of Na) during heating at 4 GPa obtained at ID27 with Paris–Edinburgh press (ESRF) [97]. (**e**) First (experimental) approximation of phase diagram of the Na-Si system in the 5–8 GPa range that reflects in situ and ex situ observations [98]. Solid circles indicate experimental observations using either ex situ (phase compositions) or in situ (temperatures) techniques.

Na_8-x_Si_46_ (structure I), Na_24-y_Si_136_ (structure II) have been first obtained under high vacuum from sodium silicide Na_4_Si_4_ [95]. However, depending on stoichiometry (i.e., x and y), nominal stability extends from negative pressures (~−4 GPa for hypothetical Si_46_ and ~−3 GPa for known metastable Si_136_) [96] to high pressures (stoichiometric Na_8_Si_46_, Na_4_Si_24_, and Na_30_._5_Si_136_ with two sodium atoms per largest clathrate cage) [93,94]. All the structures are presented in Figure 11.

In situ studies [97] (e.g., Figure 11d) allowed to observe the following features of phase transformations in Na-Si system: (i) the Na-Si equilibrium phases are Na_4_Si_4_, Na_8_Si_46_ (sI), and Na_30_Si_136_ (sII-HP), stoichiometric compounds without solid solutions; (ii) sII-HP coexists with Si-I at “low” temperatures, while sI (or Na_4_Si_24_) becomes stable at high temperature (if Si is not in excess); (iii) high Na content makes sII-HP stable up to melting temperatures and sII-HP melts congruently; (iv) co-existence of sI and sII-HP (without Si-I) is possible. Such observations are typical for in situ XRD probing of the synthesis; this information together with XRD and electrical in situ data on T_1_ [99], T_4_ [98], and T_6_ [100], allowed drawing the experimental approximation of phase diagram in the 5–8 GPa range [98] (Figure 11e).

**Figure 12:**
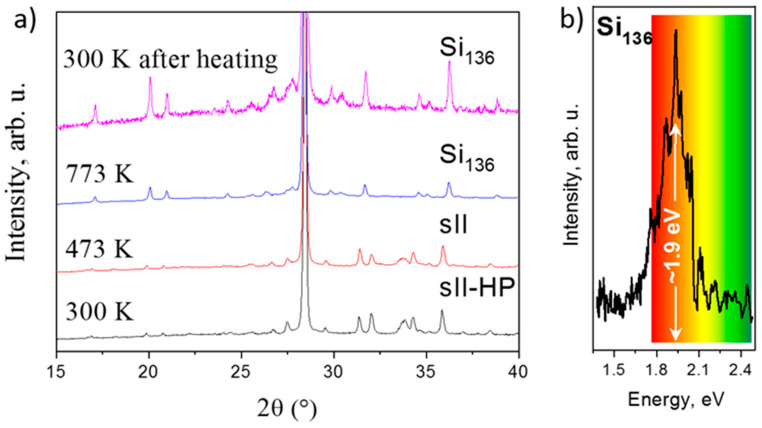
(**a**) In situ synthesis of quasi-direct bandgap allotrope of Si, Si_136_ from sII-HP clathrate performed at IMPMC laboratory using conventional diffractometry and high-temperature oven chamber (Anton Paar). Principal intensive Si-I reflection is located at ~27°. All other peaks correspond to Na_30_._5_Si_136_ at 300 K and, displaced to the left contrary expected thermal expansion, to Si_136_ at 775 K (our data). (**b**) Photoluminescence spectra of silicon allotrope with (quasi)direct bandgap c-Si_136_ in match with predicted value of quasi-direct bandgap ~2 eV (our data).

Once obtained at high pressure, Na-Si clathrates can be used as precursors for preparation of corresponding Si allotropes, two silicon frameworks remain after sodium removal, i.e., Si_24_ [14] and Si_136_. These allotropes can also, in their turn, be considered as precursors for producing other advanced silicon forms, for example Si_24_ passes into microcrystalline hexagonal silicon 4H during heating in air [101]. At our in situ example presented in Figure 12a, clathrate Na_30_._5_Si_136_ (sII-HP) loses Na atoms starting from 473 K, and passes into Si open-framework structure at ~773 K. At higher temperatures, this open-framework allotrope passes into stable Si-I phase with diamond structure. These observations illustrate quite rare “in situ” synthesis using conventional diffractometry equipped with high-temperature oven chamber HTK 1200 N (Anton Paar). This allows us to make efficient syntheses with precious high-pressure samples without synchrotron facility, at IMPMC laboratory (Paris, France).

Theoretical predictions [102] and previous optical absorption [103] and photoluminescence [104] measurements on Si_136_ and semiconductive Na_5_Si_136_ phase, respectively, confirmed a fundamental gap close to 2 eV. Figure 12b illustrates the PL signal of Si_136_ sample produced via HP-HT route, well in agreement with previous results.

## 4. Current Challenges and Future Directions

In situ high-pressure synthesis of new outstanding materials is nowadays a flourishing field of research that may lead to the patenting of many novel industrially important materials. This field is still at an initial stage but it is obvious that many discoveries will appear soon and will be useful to meet the societal challenges of the century. To this end, if the methodological tools will remain broadly similar to those mentioned in Section 2 of this review, several improvements related to the experimental and theoretical tools of the domain will open up new horizons.

Actually, while high-pressure techniques seem to be well optimized for this industrial pressure range, the constant improvement of X-ray brilliance, optics, X-ray detectors associated with the various upgrades currently being made by the 3rd generation synchrotrons will make it possible to carry out in situ synthesis experiments that will be more and more accurate and fast. Hence, various kinetics studies which are now at the limit of current X-ray technologies will be easily achievable in future years. Additionally, new possibilities opened up by the combination of diffraction and imaging or tomography under extreme conditions [48] will allow better understanding of the physical processes involved in the high-pressure synthesis of new materials. For example, recently, combined X-ray diffraction and X-ray Computed Tomography have been used to study the crystal growth kinetics of LaMn_7_O_12_ quadruple perovskite. The idea was to determine the optimized growth conditions and preferred orientations of single crystals of large size under extreme conditions. This study provides new insights regarding the growth kinetics, orientations, and crystal shape/size characteristics of the high-pressure LaMn_7_O_12_ phase, which are relevant to the field of multiferroic [105]. Such nondestructive in situ X-ray monitoring can also be used to tailor materials with outstanding mechanical or thermal properties by a quantitative microstructure analysis of transformation at high pressure and temperature, as demonstrated recently by a pioneering study on iron under extreme conditions [106].

Additionally, the progress of new precursor chemistry will take an important place in future in situ high pressure synthesis. With this development, new complex materials could become potential targets for such research. It is already the case for nanocomposites whose complex structural organization of the initial chemical reactant involves that of the ending material, synthesized in situ under high pressures and high temperatures conditions. For example, recently, the original combination of solution-phase synthesis of inorganic nanomaterials with subsequent high-pressure conditions was used to synthesize innovative nanocomposites with the in situ synchrotron probe by X-ray diffraction [107,108]. This approach paves the way to advanced multifunctional materials made of several phases not reachable at ambient conditions, which could combine the best functional properties for advanced electronic, thermal, and optical applications.

Finally, in the coming years, in situ high-pressure synthesis will also benefit from improvements of theoretical predictions which now greatly impact the ways this research can be performed. The simulations are developing with the emergence of many algorithms [83,109] and are now crucial in the design of advanced materials. These algorithms can be used not only to generate crystal structures of materials with desired outstanding properties, but also to select the most appropriate P-T conditions and paths for the materials synthesis. Therefore, before any synthesis, they can directly “guide” in situ high-pressure experiments [109]. These advances promise to optimize the process of scientific discovery of new materials by efficient ab initio-assisted in situ high-pressure synthesis. In the framework of this combined methodology, fast tests of new ideas will be possible with minimal production of waste matter as compared to classical studies.

## Data Availability

No new data were created or analyzed in this study. Data sharing is not applicable to this article.
